# Simultaneous Assessment of Rotavirus-Specific Memory B Cells and Serological Memory after B Cell Depletion Therapy with Rituximab

**DOI:** 10.1371/journal.pone.0097087

**Published:** 2014-05-12

**Authors:** Daniel Herrera, Olga L. Rojas, Carolina Duarte-Rey, Rubén D. Mantilla, Juana Ángel, Manuel A. Franco

**Affiliations:** 1 Instituto de Genética Humana, Facultad de Medicina, Pontificia Universidad Javeriana, Bogotá, Colombia; 2 Unidad de Inmunología, Escuela de Medicina y Ciencias de la Salud, Universidad del Rosario, Bogotá, Colombia; 3 Riesgo de Fractura S.A. - CAYRE I.P.S, Bogotá, Colombia; COCHIN INSTITUTE, Institut National de la Santé et de la Recherche Médicale, France

## Abstract

The mechanisms that contribute to the maintenance of serological memory are still unclear. Rotavirus (RV) memory B cells (mBc) are enriched in IgM^+^ and CD27^-^ subpopulations, which are associated with autoimmune diseases pathogenesis. In patients with autoimmune diseases treated with Rituximab (RTX), some autoantibodies (auto-Abs) decrease after treatment, but other auto-Abs and pathogen-specific IgG Abs remain unchanged. Thus, maintenance of autoimmune and pathogen-specific serological memory may depend on the type of antigen and/or Ab isotype evaluated. Antigen-specific mBc and antigen-specific Abs of different isotypes have not been simultaneously assessed in patients after RTX treatment. To study the relationship between mBc subpopulations and serological memory we characterized total, RV- and tetanus toxoid (TT)-specific mBc by flow cytometry in patients with autoimmune diseases before and after treatment with RTX. We also measured total, RV- and TT-Abs, and some auto-Abs by kinetic nephelometry, ELISA, and EliA tests, respectively. Minor differences were observed between the relative frequencies of RV-mBc in healthy controls and patients with autoimmune disease. After RTX treatment, naïve Bc and total, RV- and TT-specific mBc [IgM^+^, switched (IgA^+^/IgG^+^), IgM^+^ only, IgD^+^ only, and CD27^-^ (IgA^+^/IgG^+^/IgM^+^)] were significantly diminished. An important decrease in total plasma IgM and minor decreases in total IgG and IgA levels were also observed. IgM rheumatoid factor, IgG anti-CCP, and IgG anti-dsDNA were significantly diminished. In contrast, RV-IgA, RV-IgG and RV-IgG_1_, and TT-IgG titers remained stable. In conclusion, in patients with autoimmunity, serological memory against RV and TT seem to be maintained by long-lived plasma cells, unaffected by RTX, and an important proportion of total IgM and serological memory against some auto-antigens seem to be maintained by short-lived plasma cells, dependent on mBc precursors depleted by RTX.

## Introduction

Pathogen-specific protective IgG levels following natural infection or vaccination can persist for decades, or in some cases for a lifetime, in the apparent absence of the antigen [1]. This serological memory provides the host with a first line of defense against reinfection by many microorganisms [2], and critical pathogen-specific antibody (Ab) titers that correlate with protection have been identified for several vaccines [3]. Additionally, in autoimmune diseases, autoantibodies (auto-Abs) of different isotypes are associated with disease activity and pathogenesis [4] and in some cases predict disease severity [5]–[7].

The mechanisms that contribute to the maintenance of serological memory in healthy individuals are still unclear and, in general, have been studied only with respect to the IgG isotype and for a limited number of antigens. In healthy adults, IgG serological memory seems to be maintained by long-lived plasma cells (PC), independently of memory B cells (mBc) [1], [8]. Two non-mutually exclusive theories have been proposed to explain the survival of long-lived PC [9]: 1) long-lived PC reside in a limited number of survival niches in the bone marrow or secondary lymphoid organs. Plasmablasts may or may not gain the competence to respond to survival signals of these niches, which will finally determine their lifespan as long-lived PC or short-lived PC [8], [10]. 2) The lifespan of PC is related to the integrated signals through the B-cell receptor, which largely depend on the antigen repetitive nature, and signals obtained through CD4 T-cell help, and therefore, is imprinted at the time of the immune response induction [9]. This theory proposes that PC and mBc represent independently regulated populations [11].

However, under certain circumstances, such as autoimmunity, short-lived PC, which need to be continuously replenished by mBc, may also contribute to maintain serological memory (see below) [12]. In conditions where short-lived PC contribute to serological memory, a correlation is expected between numbers of circulating antigen-specific mBc and levels of antigen-specific serological memory [13].

Serological memory has been evaluated in patients with autoimmune diseases treated with B-cell depletion therapy using Rituximab (RTX), an anti-CD20 monoclonal Ab that depletes circulating Bc and leaves PC unaffected [14]. Given that CD20 is not expressed on PC, Bc depletion therapy with RTX allows to discriminate between the Abs secreted by short-lived PC, in turn related to mBc, and those secreted by long-lived PC [15].

After Bc depletion with one RTX cycle total IgA, IgG, IgM, and IgE levels significantly decrease, but within normal ranges [16]. In contrast, IgG Ab titers against pathogens such as measles [16], tetanus [17], and pneumococcal capsular polysaccharide [18] remain constant. In regard to auto-Abs results differ: on the one hand, it has been reported that anti-double-stranded DNA (dsDNA) and anti-C1q [18], both of IgG isotype, and IgA-, IgG-, and IgM-class rheumatoid factors (RF) diminish significantly after RTX therapy [19]. On the other hand, auto-Abs against Ro52, Ro60, and La44, also of IgG isotype, remain unchanged after RTX therapy [16]. These results suggest that the mechanism of maintenance of serological memory could depend on the type of antigen and/or Ab isotype evaluated, particularly in patients with autoimmune diseases. However, antigen-specific mBc and antigen-specific Abs of different isotypes have not been simultaneously assessed in patients that received B-cell depletion therapy with RTX.

In a recent study, we characterized circulating RV-specific B cells (RV-Bc identified by their capacity to bind GFP-coupled virus like particles [VLPs]) and tetanus toxoid-specific B cells (TT-Bc identified by their capacity to bind biotin labeled TT) and assessed their relation with serological memory in healthy volunteers [20]. Compared with TT-Bc, RV-Bc seem to be peculiar because a group of naïve Bc bind RV-VLPs [21], [22], and RV-mBc are enriched in the CD27^+^IgM^+^ (which includes the CD27^+^IgD^+^IgM^+^ subset) and in the CD27^-^IgG^+^ mBc subsets [20], [23]. In addition, CD27^+^IgA^+^ RV-mBc correlated positively with RV-IgA plasma levels, but a correlation between CD27^+^IgG^+^ RV-mBc and RV-IgG was absent. In contrast, CD27^+^IgG^+^ TT-mBc correlated with TT-IgG plasma levels. Therefore, the association between RV-mBc and serological memory seems to be somewhat different from that of TT-mBc, making it a relevant model to study the relationship between mBc and serological memory.

CD27^+^IgD^+^IgM^+^ mBc (IgM^+^ mBc) and CD27^-^ mBc, the mBc subsets in which RV-mBc are enriched, are relevant in autoimmune diseases pathogenesis. IgM^+^ mBc have been shown to be decreased in patients with systemic lupus erythematosus (SLE) [24], rheumatoid arthritis (RA) [25], and Sjögren's Syndrome [26]. Moreover, there is a negative correlation between the circulating number of IgM^+^ mBc and auto-Abs levels and disease activity in SLE patients [24]. In RA patients IgM^+^ mBc seem to migrate to the synovial membrane in a tumor necrosis factor dependent manner [25]. In contrast, circulating CD27^-^ mBc are increased in SLE patients and positively correlate with disease activity [27]. Since RV-mBc are enriched in these subsets, such cells could be related to serological memory in a unique manner and we hypothesized they could be distributed in a peculiar manner in patients with autoimmunity.

Here, total, RV- and TT-specific B-cell subsets were characterized in patients with autoimmune diseases before and after B-cell depletion therapy with RTX, and in healthy individuals. Total immunoglobulins (Igs), RV- and TT-Abs, and some auto-Abs were also measured in the same timeframes. Titers of RV-IgA, RV-IgG, and RV-IgG_1_, as well as TT-IgG, remained stable after RTX treatment. In contrast, IgM RF, IgG against cyclic cytrullinated peptide (CCP), and IgG against dsDNA were significantly diminished, as well as total Igs, especially total IgM. Thus, in patients with autoimmune diseases, serological memory against RV and TT seem to be maintained by long-lived PC, unaffected by RTX, and an important proportion of total IgM and serological memory against some auto-antigens seem to be maintained by short-lived PC, dependent on mBc depleted by RTX.

## Materials and Methods

### Ethics statement

Written informed consent was obtained from each adult volunteer. Studies were approved by the Ethics Committee of the San Ignacio Hospital and Pontificia Universidad Javeriana and conducted in accordance with the guidelines of the Helsinki Declaration.

Pools of plasma samples from children enrolled in prior published studies [28]–[30] in whom the informed consent form (also approved by the Ethics Committee of the San Ignacio Hospital and Pontificia Universidad Javeriana) authorized their use in unrelated research studies were used as positive controls in some experiments.

### Subjects

Fourteen patients, twelve females and two males, nine of them diagnosed with RA and five with SLE according to the American College of Rheumatology international criteria [31], [32] were included. All patients had moderate or high disease activity, despite treatment with disease-modifying anti-rheumatic drugs or standard immunosuppressive therapy, measured by the disease activity score of 28 joint counts (DAS28) or SLE Disease Activity Index (SLEDAI), respectively. Given the failure to standard treatment regimens, they were selected to receive RTX by their treating rheumatologist. Additional clinical manifestations considered to use RTX as the treatment of choice in patients included: lupus nephritis (n = 2), autoimmune thrombocytopenia (n = 1), overlap of RA and SLE (n = 4), and antiphospholipid syndrome (n = 2). The treatment regimen included two infusions of intravenous RTX (1,000 mg), 14 days apart, in combination with intravenous methylprednisolone (100–250 mg) [33], [34]. The median age at RTX treatment was 46 years (range 26–69) and the median disease duration to the time of RTX treatment was 3 years (range 1–30); eight patients had been diagnosed within three years or less. Clinical follow up could be done in eleven patients within the following six months post B-cell depletion therapy. Out of these, seven patients showed subjective and objective clinical improvement. Table S1 describes accompanying autoimmune diagnoses, concomitant and relevant previous pharmacologic treatment, baseline disease activity, and clinical follow up. Ten age and sex matched healthy volunteers were used as controls.

### Sample collection and processing

Peripheral blood mononuclear cells (PBMC) were isolated by LymphoSep (MP Biomedicals, Solon, OH) density-gradient centrifugation from heparinized samples from ten of the fourteen patients, described above, immediately before and four to six months after RTX infusion (in the remaining four patients PBMC samples were only available after RTX treatment), and from ten age and sex matched healthy controls. A complete blood count test was performed for each volunteer after each blood draw, and plasma was collected and stored at −80°C for subsequent total and antigen-specific Igs assessment and auto-Abs measurement.

### Production of fluorescent virus like particles (VLPs)

Fluorescent RV VLPs were a kind gift of Annie Charpilienne and were produced using baculovirus expression vectors, as previously described [35]. Briefly, Sf9 cells were co-infected with 2 recombinant baculoviruses at a multiplicity of infection greater than 5 PFU/cell. One baculovirus expressed RF (bovine RV) VP6 and the other a fusion protein consisting of green fluorescent protein (GFP) fused to the N terminus of RF VP2 deleted in the first 92 amino acids. Infected cultures were collected five to seven days post infection and purified by density gradient centrifugation in CsCl. The optimal concentration of the RV VLPs for labeling of specific mBc was determined using PBMC from healthy volunteers. Of note, RV VP6 is an immunodominant protein, and the majority of human RV-specific B cells bind to VP6 [36]. Additionally, the majority of RV antibodies in infected animals and humans recognize the VP6 protein on the outer shell of the VLPs [37].

### Biotinylated - TT antigen

Tetanus Toxoid (Statens Serum Institute, Denmark) for *in vitro* tests was biotinylated using the EZ-Link Photoactivatable Biotin kit (Pierce Biotechnology, Rockford, IL) according to manufacturer's instructions, as previously described [20], with minor modifications. A total of 750 µg of TT protein was incubated with biotin at a molar ratio of 20 mol of dye per mole of protein, for 20 min on ice under a UV (365 nm) lamp, and then dialyzed against PBS for 18 hours, to remove excess biotin, using a Slide-A-Lyzer dialysis cassette (Thermo Scientific Pierce, Illkirch, France). The optimal concentration of the biotinylated TT for labeling of specific mBc was determined using PBMC from healthy and recently TT vaccinated volunteers, and the specificity of binding was evaluated with a competition assay using non-biotinylated TT (data not shown).

### Flow cytometry assays

Fresh PBMC, 4 to 6×10^6^, were washed twice with PBS (Gibco-BRL, Gaithersburg, MD) and incubated with the GFP labeled VLPs (0.9 µg/test) or without this reagent (negative control) for 45 minutes in the dark, at room temperature (RT). The cells were then washed with PBS – 1% bovine serum albumin (Merck, Darmstadt, Germany), 0.02% sodium azide (Mallinckrodt Chemicals, Paris, KY) (staining buffer), and surface stained with Abs against CD19-allophycocyanin (APC)-H7 (SJ25C1 clone; Becton Dickinson [BD], San Jose, CA), IgD-Horizon V450 (IA6-2 clone; BD), CD27- phycoerythrin (PE)-Cy7 (M-T271 clone; BD), goat anti-IgA-R-PE (Jackson ImmunoResearch, West Grove, PA), IgG-APC (G18-145 clone; BD) and custom-made IgM-Alexa Fluor 700 (145-8 clone; BD). Biotinylated-TT, or no reagent, was also added at this step and incubated for 30 minutes in the dark, at RT. Cells were then washed with staining buffer and the biotinylated-TT was detected using streptavidin-peridinin chlorophyll protein (PerCP) (BD). Streptavidin-PerCP was also added to the PBMC without biotinylated-TT to assess the background generated by this reagent (negative control). After staining, the cells were washed and resuspended in staining buffer. At least 200,000 B cells, gated on a CD19^+^ window, were acquired on a FACSAria (BD) or LSRFortessa (BD) flow cytometer. CD19 was used as the marker to recognize total B cells since CD20 is rapidly lost from RTX-coated B cells [38]. Application settings were used to obtain constant fluorescence intensity values among experiments run on different days and regardless of the flow cytometer used. Fluorescence minus one controls were used to determine the cut-off between positive and negative cell populations for each marker [39]. Of note, Abs against CD3/CD14-Horizon V500 (UCHT1 and M5E2 clones, respectively; BD) were used as a dump channel in samples taken from patients after RTX treatment. However, since conclusions were similar with or without its use, the dump channel was disregarded in the final analyses.

Results for total and antigen-specific mBc were expressed as absolute numbers of CD19^+^ B cells/mL, calculated after background subtraction, based on the total lymphocyte numbers in the patient's complete blood count test results.

Evaluable total B cell subpopulations were defined as those with ≥10 acquired events [40]. The frequency, in terms of CD19^+^ B cells, of those 10 events was calculated considering the median CD19^+^ events acquired after RTX treatment, and multiplied by the median absolute B cell count after RTX treatment. This value, 3.5 CD19^+^ B cells/mL, represents the flow cytometry detection limit for total B cell subpopulations. A common detection limit could not be determined for antigen-specific mBc subsets due to the variable background. Nevertheless, the reported values correspond to antigen-specific mBc subsets with ≥10 acquired events, and at least two-fold greater than the observed background.

Flow cytometry analysis was performed using FlowJo software version 9.6.2 for Mac (Treestar, Ashland, OR).

### Measurement of total immunoglobulins (IgA, IgG and IgM) and IgM Rheumatoid Factor

Plasma samples were thawed and simultaneously assessed by kinetic nephelometry on an IMMAGE immunochemistry system (Beckman Coulter, Fullerton, CA), following the manufacturer's instructions. Measuring ranges are as follows: IgA: 40–700 mg/dL (normal reference range values: 82–453 mg/dL); IgG: 200–3600 mg/dL (normal reference range values: 751–1560 mg/dL); IgM: 25–400 mg/dL (normal reference range values: 46–304 mg/dL); and IgM rheumatoid factor (RF): 20–800 IU/mL (negative cut off value: <20 IU/mL), with a sensitivity level in RA patients reported by the manufacturer of 70–75%.

### ELISAs for detection of RV-specific IgA, IgG, and IgG1 and TT-specific IgG in plasma

RV and TT antibodies were assessed as previously described with minor modifications [20]: 96-well vinyl microtiter ELISA plates (Thermo Electron Corporation, Milford, MA) were coated with either a supernatant from RF virus-infected MA104 cells (cell line established from an African green monkey fetal kidney highly susceptible to RV infection) or the supernatant of mock-infected cells (negative control) [41], for RV-specific ELISAs, or 0.5 µg/mL of TT or PBS (negative control), for TT-specific IgG ELISA, and incubated overnight at 4°C. After blocking, serial dilutions of plasma samples were deposited in each well. After incubation, the following sequence of reagents was added: biotin-labeled goat anti-human IgA or IgG (Kirkegaard & Perry Laboratories [KPL], Gaithersburg, MD) or, biotin-labeled mouse anti-human IgG_1_ (Sigma-Aldrich, St. Louis, MO); streptavidin-peroxidase (KPL) and tetramethyl benzidine substrate (KPL). Pools of plasma samples from children with RV-IgA, or RV-IgG, and from adults with TT-IgG were used as positive controls. Plasmas from a child without evidence of previous RV infection (RV-IgA^-^) and from an adult negative for TT-IgG were used as negative controls. Samples were considered positive if the optical density in the experimental wells was >0.1 units and two-fold greater than the optical density in the corresponding negative control wells. To be accepted for analysis, the titer of the positive control plasma could not differ by more than one dilution from plate to plate.

### Measurement of anti-CCP and anti-dsDNA autoantibodies (IgG isotype)

Plasma samples were simultaneously assessed by a fluorescence enzyme immunoassay (EliA test), following the manufacturer's instructions, on a Phadia ImmunoCAP 100 system (Phadia AB, Uppsala, Sweden). The anti-CCP test has a measuring range of 0.4 to at least 340 U/mL, a negative cut off value of <7 U/mL, an equivocal range of 7–10 U/mL, and a positive cut off value of >10 U/mL; the manufacturer's reported clinical sensitivity and specificity are 87.8% and 96.7%, respectively, in RA patients. The anti-dsDNA test has a measuring range of 0.5 to at least 400 IU/mL, a negative cut off value of <10 IU/mL, an equivocal range of 10–15 IU/mL, and a positive cut off value of >15 IU/mL; the manufacturer's reported sensitivity for active SLE is 70.8% and clinical specificity of 93.2%.

### Statistical analyses

Analysis was performed with SPSS software version 20.0 (IBM Inc.) and with GraphPad Prism version 6. Differences between groups were evaluated with nonparametric Mann–Whitney and Wilcoxon tests, as required. Correlations were evaluated with Spearman's test. When data followed a normal distribution, a Student's *t*-test was applied. Significance was established if *p*<0.05, in 2 tailed tests.

## Results

### RV-mBc are enriched in the circulating IgM^+^ mBc subsets in both controls and patients

**Figure 1:**
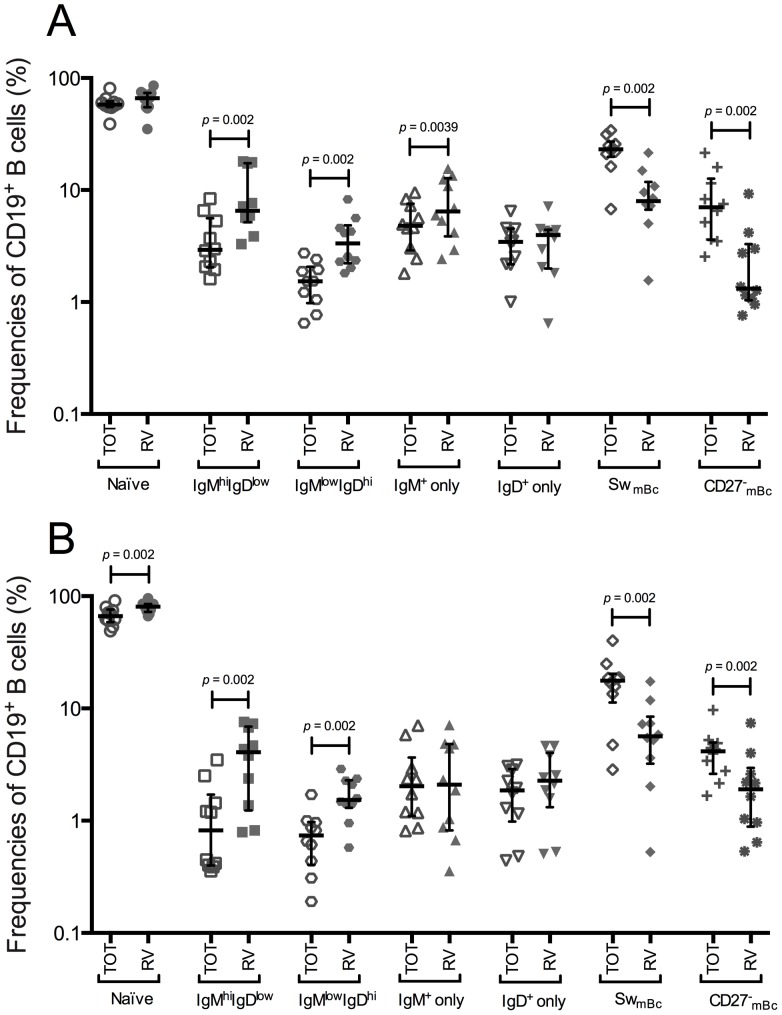
RV-mBc are enriched in the IgM^hi^IgD^low^, IgM^low^IgD^hi^, and IgM^+^ only mBc subsets. Summary of the frequencies of seven subsets of total and RV-mBc assessed by multiparametric flow cytometry. All *p* values reported are 2-tailed (*p*<0.05, Wilcoxon test). Lines and error bars denote the median and interquartile range, respectively. **A**. Healthy volunteers (n = 10). **B**. Patients (n = 10) with autoimmune diseases before RTX treatment.

Since patients with RA and SLE show altered frequencies in the IgM^+^ mBc and CD27^-^ mBc subpopulations [24], [25], [27], in which RV-mBc are enriched, we compared the relative frequencies of total and RV-Bc subsets in healthy adult volunteers (HV) and patients prior RTX treatment (prior-RTX) by flow cytometry (see analysis strategy in Figure S1A). As expected [36], an enrichment of RV-mBc was found in the IgM^hi^IgD^low^ subset in HV and patients prior-RTX (Figure 1A and 1B, respectively). Additionally, an enrichment of RV-mBc was also found in the IgM^low^IgD^hi^ mBc subset in both HV and patients prior-RTX, and in the IgM^+^ only mBc subset in HV (Figure 1A). An apparent enrichment of RV-naïve Bc was observed in patients prior-RTX (Figure 1B).

An enrichment of RV-mBc was not detected in the CD27^-^ mBc subset in HV and patients prior-RTX (Figure 1A and 1B, respectively). However, in agreement with previous results [20], when the analysis was done in terms of the CD27^-^ mBc, RV-mBc were enriched in the CD27^-^IgG^+^ subset in HV, but not in patients prior-RTX (data not shown).

Thus, the relative contribution of each mBc subset to RV-mBc was, in general, similar between HV and patients prior-RTX.

### Total and antigen-specific Bc subsets are significantly decreased after B-cell depletion therapy with RTX

Patients with autoimmune diseases treated with RTX represent a unique opportunity to assess the relation between mBc and serological memory in humans, because it provides a scenario in which one of the components, circulating mBc, is temporarily absent, so its effect on the other component, serological memory, can be evaluated. Therefore, we compared the absolute numbers (CD19^+^ B cells/mL) of total, RV- and TT-specific Bc subsets in ten HV and ten patients before and after RTX treatment (see analysis strategy and a representative flow cytometry plot in Figure S1B and Figure S2 for a HV and a patient assessed four months after RTX treatment, respectively).

**Figure 2:**
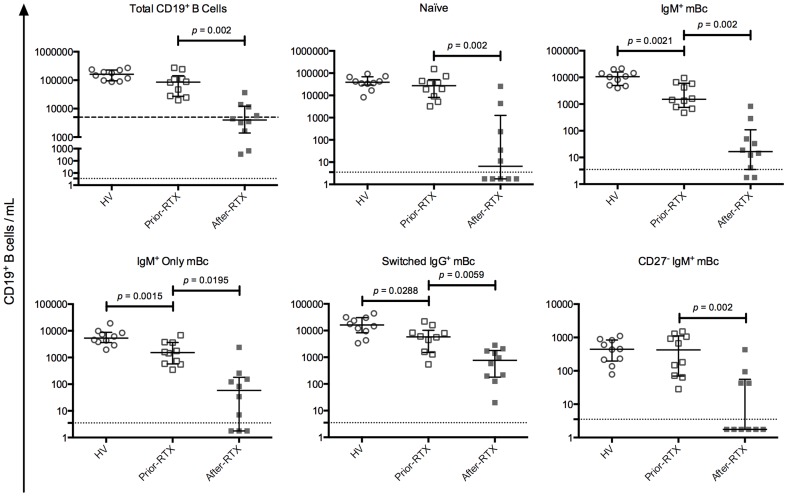
Comparison of selected total B cell subpopulations among the study groups. Healthy volunteers (HV) (n = 10), patients before RTX treatment (prior-RTX) (n = 10) and patients after RTX treatment (after-RTX) (n = 10). For total CD19^+^ B cells, the dashed line represents the clinical depletion limit after RTX treatment (less than 5,000 CD19^+^ cells/mL). The dotted lines represent the estimated flow cytometry detection limit of 3.5 CD19^+^ B cells/mL. Solid lines and error bars denote the median and interquartile range, respectively. Differences between HV and patients prior-RTX were evaluated with Mann–Whitney tests and between patients prior-RTX and patients after-RTX with Wilcoxon tests. All *p* values reported are 2-tailed.

First, we compared circulating total mBc subsets present in HV and patients prior-RTX. As shown in Figure 2, IgM^+^ mBc, IgM^+^ only mBc, and switched IgG^+^ mBc were significantly decreased in patients prior-RTX, compared with HV. The same was observed for IgM^+^ mBc IgM^hi^IgD^low^ and IgM^low^IgD^hi^, IgD^+^ only mBc, and switched IgA^+^ mBc (see Figure S3).

Second, we compared circulating total mBc subsets present in patients before and after-RTX. After RTX treatment, six of the ten patients studied had clinical peripheral Bc depletion (less than 5,000 CD19^+^ cells/mL) (Figure 2). The four patients assessed by flow cytometry only after RTX treatment also had clinical peripheral Bc depletion (data not shown). In patients after-RTX a significant decrease in all total Bc subsets was observed (Figure 2 and Figure S3). The median decrease in all subsets, with respect to the pretreatment values, was above 96%, except for switched IgA^+^ and switched IgG^+^ mBc, which had a median decrease of 87% (data not shown).

**Figure 3:**
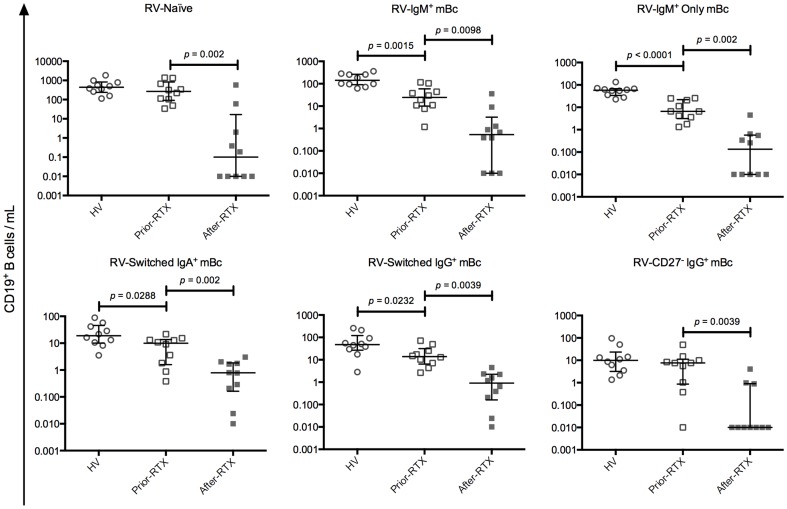
Comparison of RV-specific B cell subpopulations among the study groups. Solid lines and error bars denote the median and interquartile range, respectively. Differences between HV (n = 10) and patients prior-RTX (n = 10) were evaluated with Mann–Whitney tests and between patients prior-RTX and patients after-RTX (n = 10) with Wilcoxon tests. All *p* values reported are 2-tailed.

Third, we compared circulating antigen-specific mBc subsets present in HV and patients before and after-RTX. RV-IgM^+^ mBc, RV-IgM^+^ only mBc, RV-switched IgA^+^ mBc, and RV-switched IgG^+^ mBc were significantly decreased in patients prior-RTX, compared with HV (Figure 3). In addition, RV-IgM^+^ mBc IgM^hi^IgD^low^ and IgM^low^IgD^hi^, and RV-IgD^+^ only mBc were also significantly diminished (see Figure S4). In patients after-RTX a significant decrease in all RV- and TT-specific Bc subsets was observed (Figure 3 and Figure S4). The median decrease in all subsets, respect to the baseline values, was above 98%, except for RV-switched IgA^+^ and RV-switched IgG^+^ mBc, which had a median decrease of 92% and 93%, respectively.

Taken together, these results showed that in patients prior-RTX total and antigen-specific-mBc subsets decreased in similar proportions. Of note, we confirmed that patients prior-RTX have decreased IgM^+^ mBc subsets and switched mBc [24], [42]. In patients after-RTX both total and antigen-specific Bc subsets are significantly diminished at similar levels.

### Total plasma immunoglobulins and auto-Abs, unlike RV- and TT-Abs, are significantly decreased after B-cell depletion therapy with RTX

**Figure 4:**
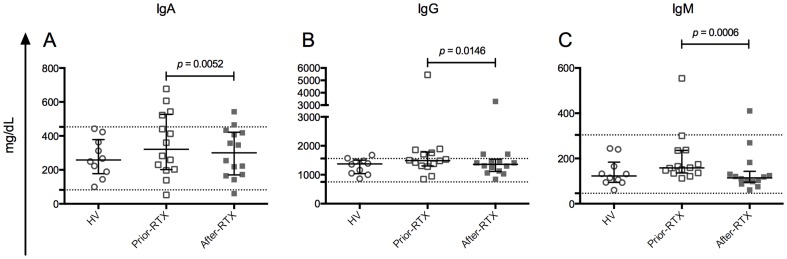
Comparison of total immunoglobulins in plasma among the study groups. Levels of total IgA (**A**), IgG (**B**) and IgM (**C**) in plasma determined by kinetic nephelometry. The dotted lines represent the lower and upper values of the normal reference range (IgA: 82–453 mg/dL; IgG: 751–1560 mg/dL; IgM: 46–304 mg/dL). Solid lines and error bars denote the median and interquartile range, respectively. Differences between HV (n = 10) and patients prior-RTX (n = 14) were evaluated with Mann–Whitney tests and between patients prior-RTX and patients after-RTX (n = 14) with Wilcoxon tests. All *p* values reported are 2-tailed.

To determine the effect of B-cell depletion therapy with RTX on the total serological memory, we measured the levels of total plasma IgA, IgG, and IgM by kinetic nephelometry (Figure 4). Levels of total plasma Igs in patients prior-RTX and HV were similar; nevertheless, total IgM tended to be higher in patients prior-RTX (2-tailed Mann-Whitney's test, *p* = 0.0578). As expected [16], after RTX treatment total plasma Igs were significantly decreased, although remained within normal ranges (Figure 4). Two patients had an increase in the IgA level, one of them also in the IgM level, and another patient showed an increase in the IgG level. Of note, excluding the patients who showed an increase after RTX treatment, the total plasma IgM percentage decrease, respect to the pretreatment values, was significantly greater than the percentage decrease for total IgA and IgG (2-tailed Student's *t*-test, *p* = 0.0031 and *p* = 0.0093, respectively) (data not shown).

**Figure 5:**
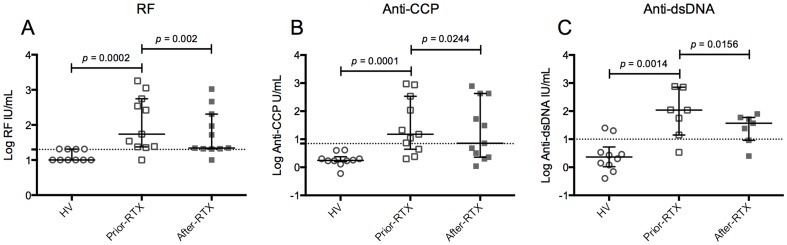
Comparison of RF, anti-CCP and anti-dsDNA autoantibodies levels in plasma among the study groups. Levels of rheumatoid factor (RF) determined by kinetic nephelometry (**A**), anti-cyclic citrullinated peptide (anti-CCP) (**B**) and anti-doubled stranded DNA (anti-dsDNA) (**C**) determined by a fluorescence enzyme immunoassay (EliA test) in patients diagnosed with RA (**A** and **B**) (n = 11) or with SLE (**C**) (n = 7) (some patients had both diagnoses). Solid lines and error bars denote the median and interquartile range, respectively. The dotted lines correspond to the clinical limits below which a sample is considered negative according to the technique used for its detection (RF: <20 IU/mL, anti-CCP: <7 U/mL and anti-dsDNA: <10 IU/mL). Differences between HV and patients prior-RTX were evaluated with Mann–Whitney tests and between patients prior-RTX and patients after-RTX with Wilcoxon tests. All *p* values reported are 2-tailed.

To evaluate the effect of B-cell depletion therapy with RTX on autoimmune serological memory, we assessed IgM-RF and IgG-anti-CCP levels (relevant auto-Abs in RA) and IgG-anti-dsDNA levels (relevant auto-Ab in SLE) in patients with the corresponding diagnoses before and after RTX treatment, and in HV as controls. In four HV the levels of RF were just above the negative cut off value (Figure 5A), in all of them levels of anti-CCP auto-Abs were negative (Figure 5B), and in two levels of anti-dsDNA were positive (Figure 5C). Compared with HV, patients prior-RTX had higher RF, anti-CCP, and anti-dsDNA auto-Abs levels (Figure 5A, 5B, and 5C, respectively). Following RTX treatment, all auto-Abs levels were significantly diminished (Figure 5).

In order to contrast the effect of B-cell depletion therapy on the levels of individual auto-Abs and their respective isotype of total plasma Ig, data after RTX treatment were expressed as the percentage decrease respect to the pretreatment values. Only patients with an abnormal pretreatment value were analyzed. The percentage drop in IgG-anti-dsDNA and IgG-anti-CCP was significantly higher than that in total plasma IgG (2-tailed Student's *t*-test, *p*<0.0001 and *p* = 0.0378, respectively). There was no significant difference between the percentage drop in IgM-RF and total plasma IgM (data not shown).

**Figure 6:**
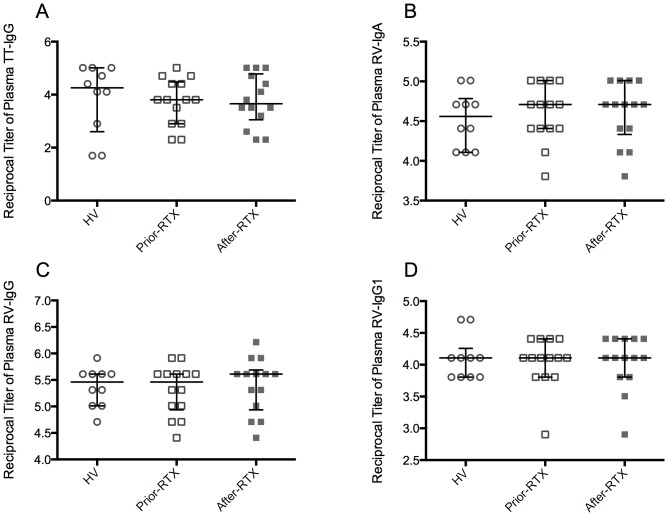
Comparison of RV- and TT-specific immunoglobulins titers in plasma among the study groups. Titers of TT-IgG (**A**), RV-IgA (**B**), RV-IgG (**C**) and RV-IgG_1_ (**D**) in plasma determined by ELISA. Solid lines and error bars denote the median and interquartile range, respectively. Differences between HV (n = 10) and patients prior-RTX (n = 14) were evaluated with Mann–Whitney tests and between patients prior-RTX and patients after-RTX (n = 14) with Wilcoxon tests.

As expected [17], and in contrast to the effect of B-cell depletion therapy on total plasma Igs and the auto-Abs evaluated, plasma TT-IgG titers remained constant after RTX treatment (Figure 6A). Similarly, RV-IgA (Figure 6B) and RV-IgG (Figure 6C) remained unchanged. We further assessed the RV-IgG_1_ titers because CD27^-^IgG^+^ mBc predominantly express the IgG_1_ and IgG_3_ subclasses [43], RV-mBc are enriched in the CD27^-^IgG^+^ subset, and RV-IgG_1_ seems to be a predominant RV-IgG subclass [44]. Plasma RV-IgG_1_ titers in patients before and after RTX treatment were similar (Figure 6D).

In conclusion, B-cell depletion therapy with RTX did not have an effect on pathogen-specific serological memory, irrespective of the isotype evaluated, but significantly decreased the auto-Abs assessed and the total plasma Igs, especially total IgM.

### Correlations between mBc subsets and serological memory

We next set to determine if selected mBc subsets could correlate with total Igs, auto-Abs or Ag-specific Abs. All IgM^+^ mBc subsets correlated with total plasma IgM (Table 1) only when data before and after RTX treatment in all patients were analyzed jointly. Likewise, in patients with SLE as their principal diagnosis, total switched IgG^+^ and CD27^-^IgG^+^ mBc correlated with anti-dsDNA levels.

**Table 1:** Memory B cell subsets that correlate with total immunoglobulins.

Cell subset^a^	*P* Value^b^	*Rho*
**Pt. IgM^+^ Only mBc with total plasma IgM**	0.0013	0.6687
**Pt. IgM^+^ mBc with total plasma IgM**	0.0305	0.4842
**Pt. IgM^+^ mBc IgM^hi^ IgD^low^ with total plasma IgM**	0.0148	0.5361
**Pt. IgM^+^ mBc IgM^low^ IgD^hi^ with total plasma IgM**	0.0306	0.484
**Pt. CD27^-^ IgM^+^ mBc with total plasma IgM**	0.0364	0.4702

a All correlations for patients (Pt.) were established when data before and after RTX treatment were analyzed jointly (n = 10).

b Spearman's two-tailed test.

TT-switched IgG^+^ mBc correlated with plasma TT-IgG titers in HV; in contrast, correlations between RV-switched IgA^+^ and IgG^+^ mBc and their corresponding plasma RV-IgA or RV-IgG titers were absent in HV and in patients (Table 2 and data not shown).

**Table 2:** Memory B cell subsets that correlate with antigen-specific immunoglobulins or autoantibodies.

Cell subset^a^	*P* Value^b^	*Rho*
**HV. TT-Switched IgG^+^ mBc with TT-IgG**	0.0242	0.7184
**SLE Pt. Switched IgG^+^ mBc with anti-dsDNA**	0.0438	0.6606
**SLE Pt. CD27^-^ IgG^+^ mBc with anti-dsDNA**	0.0234	0.7212

a All correlations for patients (Pt.) were established when data before and after RTX treatment were analyzed jointly (n = 10).

b Spearman's two-tailed test.

## Discussion

Here, we show that despite a significant decrease of circulating RV- and TT-specific mBc after B-cell depletion therapy with RTX (Figure 3 and Figure S4), plasma RV- and TT- Abs remained constant (Figure 6). In contrast, the auto-Abs measured were significantly diminished (Figure 5). Similarly, total IgM decreased at significantly higher levels than total IgA and IgG (Figure 4 and data not shown). Moreover, when data before and after RTX treatment were analyzed jointly, IgM^+^ mBc subsets and total plasma IgM correlated (Table 1). To the best of our knowledge, this is the first time that circulating antigen-specific mBc and antigen-specific Abs of the IgA and IgG_1_ isotypes are determined in patients receiving B-cell depletion therapy with RTX, and the first time that a correlation between several IgM^+^ mBc subsets and total plasma IgM is described in patients with autoimmunity.

The results presented here advance our knowledge of RV-mBc. We confirmed [20], [23], [36] that in HV RV-mBc are enriched in the IgM^hi^IgD^low^ subset and found that RV-mBc were also significantly enriched in the IgM^low^IgD^hi^ and IgM^+^ only mBc subsets (Figure 1A), a tendency previously observed [36]. In contrast, in patients prior-RTX enrichment of RV-mBc in the IgM^+^ only mBc subset (Figure 1B) was absent, possibly because this subset was significantly decreased compared with HV (Figure 3). An enrichment of RV-naïve Bc was observed in patients prior-RTX (Figure 1B). However, this may be simply because total and RV-IgM^+^ mBc subsets were significantly diminished in patients prior-RTX compared with HV (Figure 2), or because naïve autoreactive B cells in patients with autoimmune diseases may be more susceptible to cross-react with other antigens (due to a deficiency in negative selection) [45].

The enrichment of RV-mBc in all IgM^+^ mBc subsets in HV is intriguing. IgM^+^ mBc are heterogeneous: the IgM^hi^IgD^low^ subset resembles the spleen marginal zone B cells, may use a prediversified subset of Ig genes and participates in “innate” Ig responses to pathogens [46]. However, other IgM^+^ mBc subsets seem to behave as “true” antigen selected mBc [47]. Similarly, the enrichment of RV-mBc in the IgM^+^ only subset (at the expense of switched IgA^+^ or IgG^+^ mBc), which has a lower frequency of somatic mutations [23], suggests that RV-mBc have undergone a less extensive maturation in germinal centers than other mBc subpopulations. Besides, in an adoptive transfer immunodeficient mice model, human CD27^+^IgM^+^ RV-mBc switch to IgG *in vitro* and *in vivo*, and significantly reduce RV antigenemia and viremia [36]. Thus, further studies are required to understand the repertoire of IgM^+^ RV-mBc.

The response to RTX treatment is variable; it usually induces a very significant reduction of circulating Bc subpopulations for six to nine months after one cycle of therapy [14]. Ten of the fourteen patients studied had clinical peripheral Bc depletion by RTX (Figure 2 and data not shown). Levels of circulating Bc in the other four patients were also significantly diminished (Figure 2). Blood samples from two of these four patients were taken 5 and 6 months after RTX treatment, respectively. Therefore, it is possible that RTX therapy had failed to completely deplete them of circulating Bc or that they were already in the repopulation phase post treatment. Regardless of the case, our conclusions are unaffected by these findings.

Information on the susceptibility to depletion by RTX of pathogen-specific memory B cells subsets in different tissues in humans is scarce. A study in which three patients were splenectomized 3, 6, and 15 months after RTX treatment, vaccinia-specific memory B cells were significantly reduced in the spleen of the first two patients, with a very low frequency of B cells (around 0.1%), for the first one in the apparent absence of circulating B cells [48]. Therefore, we cannot rule out the possibility that some RV- and TT- memory B cells may survive RTX treatment in primary or secondary lymphoid organs. However, studies in primates showed that although higher doses of RTX are required to deplete bone marrow, spleen, and lymph nodes, B cell depletion in these organs is significant [49], [50]. Thus, we consider that partial, but significant, tissue RV- or TT- B cell depletion by RTX does not affect our conclusions, because in the same patients were auto-Abs evaluated decreased, RV or TT-Abs titers were unchanged.

Concerning serological memory, altogether our results suggest that at least a subset of IgM-RF, IgG-anti-CCP, and IgG-anti-dsDNA auto-Abs are mainly maintained by short-lived PC, which are probably in equilibrium with auto-Ag-specific mBc depleted by RTX. In contrast, long-lived PC, unaffected by RTX treatment, probably maintain serological memory against pathogens, like TT and RV, regardless of the antigen-specific isotype and subclass studied here. The small effect of B-cell depletion therapy on total IgA and IgG (Figure 4) probably reflects its differential effect on both auto-Abs and Abs against pathogens.

Here, we present direct evidence that in the absence of circulating TT- and RV- mBc the corresponding antigen-specific serological memory remains steady, at least 4 – 6 months after RTX treatment, and therefore seems to be maintained by long-lived PC unaffected by RTX. In agreement with this finding, in some mouse models antigen-specific serological memory is maintained by long-lived PC [51]–[53]. These and other findings [11], [54], [55] are at odds with the mBc bystander activation model of the maintenance of serological memory in healthy individuals [12]. The existence of correlations between mBc and serological memory in healthy individuals has been put forward to support this model [12]. However, antigen-specific mBc correlate with serum antigen-specific Ab levels only in some cases [1], and these correlations do not necessarily imply a cause-and-effect relationship between the mBc and the corresponding Abs. Accordingly, although TT-switched IgG^+^ mBc correlated with plasma TT-IgG titers in HV (Table 2), these titers were stable after RTX treatment; thus, the latter correlation may simply reflect equally stable but independently maintained parameters [1], [11].

The lack of correlation between RV-Abs and RV-mBc may be related to the fact that the GFP-VLPs used to measure the RV-mBc, because of their antigen repetitive nature and high avidity, can detect low affinity Bc [23] that produce antibodies undetected in the RV-ELISAs. Furthermore, a lack of correlation between mBc and serological memory does not necessarily indicate maintenance of serological memory by long-lived PC. In our patients with autoimmunity, all auto-Abs studied seem to be mainly maintained by short-lived PC, but only a correlation between switched IgG^+^ mBc and CD27^-^ IgG^+^ mBc with anti-ds-DNA auto-Abs in SLE patients was found (Table 2). However, this last correlation, and the lack of other expected correlations, must be analyzed with caution, because direct assessment of auto-antigen-specific mBc and the corresponding auto-Abs is lacking.

As previously shown for IgG-RF and IgG-anti-CCP auto-Abs [19], we observed that the percentage drops in IgG-anti-dsDNA and IgG-anti-CCP were significantly higher than the percentage drops in total plasma IgG. These results suggest a differential effect of B-cell depletion therapy with RTX on the various types of IgG serological memory. Similarly, when IgA-RF and total IgA were assessed after B-cell depletion therapy with RTX, the decrease of the IgA-RF was significantly greater than that in total serum IgA [19].

In contrast to circulating total IgG and IgA, a high proportion of the total IgM pool may depend on short-lived PC, because the decrease effect of B-cell depletion therapy with RTX on IgM-RF and total plasma IgM was similar in this and prior studies [19]. Accordingly, all IgM^+^ mBc subsets correlated with total plasma IgM when data before and after RTX treatment were analyzed jointly (Table 1). Furthermore, total IgM levels have been reported to decrease more rapidly than total IgA and IgG after B-cell depletion therapy with RTX [16]. Of note, in patients with autoimmune diseases the total IgM pool may be enriched in Abs produced by short-lived PC, since the overexpression of IL-10 and CD154, detected in some patients, have been associated to a rapid PC differentiation and auto-Ab production [56]–[58]. Plasma RV-IgM was undetected in all volunteers (data not shown); thus, the effect of B-cell depletion therapy on pathogen-specific plasma IgM remains undetermined.

Although total plasma IgM tended to be higher in patients prior-RTX (2-tailed Mann-Whitney's test, *p* = 0.0578) (Figure 4) all IgM^+^ mBc subsets were significantly diminished compared with HV (Figure 2 and Figure S3). Even so, IgM^+^ mBc subsets correlated with total plasma IgM when data before and after RTX treatment were analyzed jointly (Table 1). Thus, it is possible that PC producing autoimmune IgM, localized in ectopic lymphoid structures, may be related to circulating IgM^+^ mBc subsets and account for an important proportion of the total circulating IgM Abs. In support of this hypothesis, IgM^+^ mBc (CD27^+^IgD^+^IgM^+^) seem to migrate to the synovium in RA patients [25], and its decreased frequency negatively correlates with auto-Abs levels and disease activity in SLE patients [24]. Moreover, in a human TNF transgenic mouse model of RA, pathogenic B cells accumulate in lymph nodes draining arthritic joints and are eliminated after B-cell depletion therapy with RTX, with a concomitant disease improvement [59].

## Conclusions

In patients with autoimmunity serological memory against TT and RV, irrespective of the isotype and subclass studied here, seems to be maintained by long-lived PC, unaffected by B-cell depletion therapy with RTX. In contrast, short-lived PC, continuously replenished by mBc, seem to maintain some auto-Abs and an important proportion of IgM Abs.

## Supporting Information

Figure S1 RV-memory B cells gating strategies. Two comparable analysis strategies were used to dissect total and RV-Bc subsets (CD19^+^) based on their expression of surface markers. A representative result is shown for a HV. Naïve and three main subsets of mBc can be identified based on IgD and CD27 expression: naïve Bc (IgD^+^CD27^−^), IgM^+^ mBc (IgD^+^CD27^+^), switched mBc (IgD^−^CD27^+^), and CD27^−^ mBc (IgD^−^CD27^−^). **A**. If only CD27^+^ mBc are considered (naïve and CD27^−^ mBc are excluded) five mBc subsets can be defined in terms of IgM and IgD expression: IgM^+^ only mBc, IgM^+^ mBc IgM^hi^IgD^low^, IgM^+^ mBc IgM^low^IgD^hi^, IgD^+^ only mBc, and switched mBc (IgM^−^IgD^−^). Total (top row plots) and RV-mBc (VLPs-GFP^+^) (lower row plots) were gated. **B**. When isotype expression is considered, naïve B cells and IgM^+^ mBc express IgD and IgM; switched mBc express IgG and IgA, but a subset which only expresses IgM can also be identified on the IgD^−^CD27^+^ gate: IgM^+^ only mBc; and CD27- mBc express IgA, IgG or IgM.
(TIFF)

Figure S2 RV-memory B cells gating strategies after RTX treatment. A representative result is shown for a patient four months after RTX treatment. The gating strategy is the same as the one presented in Figure S1B. The number of acquired events per window is shown in addition to the percentage per subset.
(TIFF)

Figure S3 Comparison of other total B cell subpopulations among the study groups. Other total B cell subpopulations studied are shown for healthy volunteers (HV), patients before RTX treatment (prior-RTX) and patients after RTX treatment (after-RTX). The dotted lines represent the estimated flow cytometry detection limit of 3.5 CD19^+^ B cells/mL. Solid lines and error bars denote the median and interquartile range, respectively. Differences between HV (n = 10) and patients prior-RTX (n = 10) were evaluated with Mann–Whitney tests and between patients prior-RTX and patients after-RTX (n = 10) with Wilcoxon tests. All *p* values reported are 2-tailed.
(TIFF)

Figure S4 Comparison of other RV and TT-specific B cell subpopulations among the study groups. Other RV- and TT-specific B cell subpopulations studied are shown for HV (n = 10), patients prior-RTX (n = 10) and patients after-RTX (n = 10). Solid lines and error bars denote the median and interquartile range, respectively. Differences between HV and patients prior-RTX were evaluated with Mann–Whitney tests and between patients prior-RTX and patients after-RTX with Wilcoxon tests. All *p* values reported are 2-tailed.
(TIFF)

Table S1 Clinical features of patients with autoimmune diseases.
(DOCX)
