# Supplementary figures and images for: Simultaneous Assessment of Rotavirus-Specific Memory B Cells and Serological Memory after B Cell Depletion Therapy with Rituximab
Source: PLoS One. 2014 May 12;9(5):e97087. doi: 10.1371/journal.pone.0097087 (PMC4018270; doi:10.1371/journal.pone.0097087)

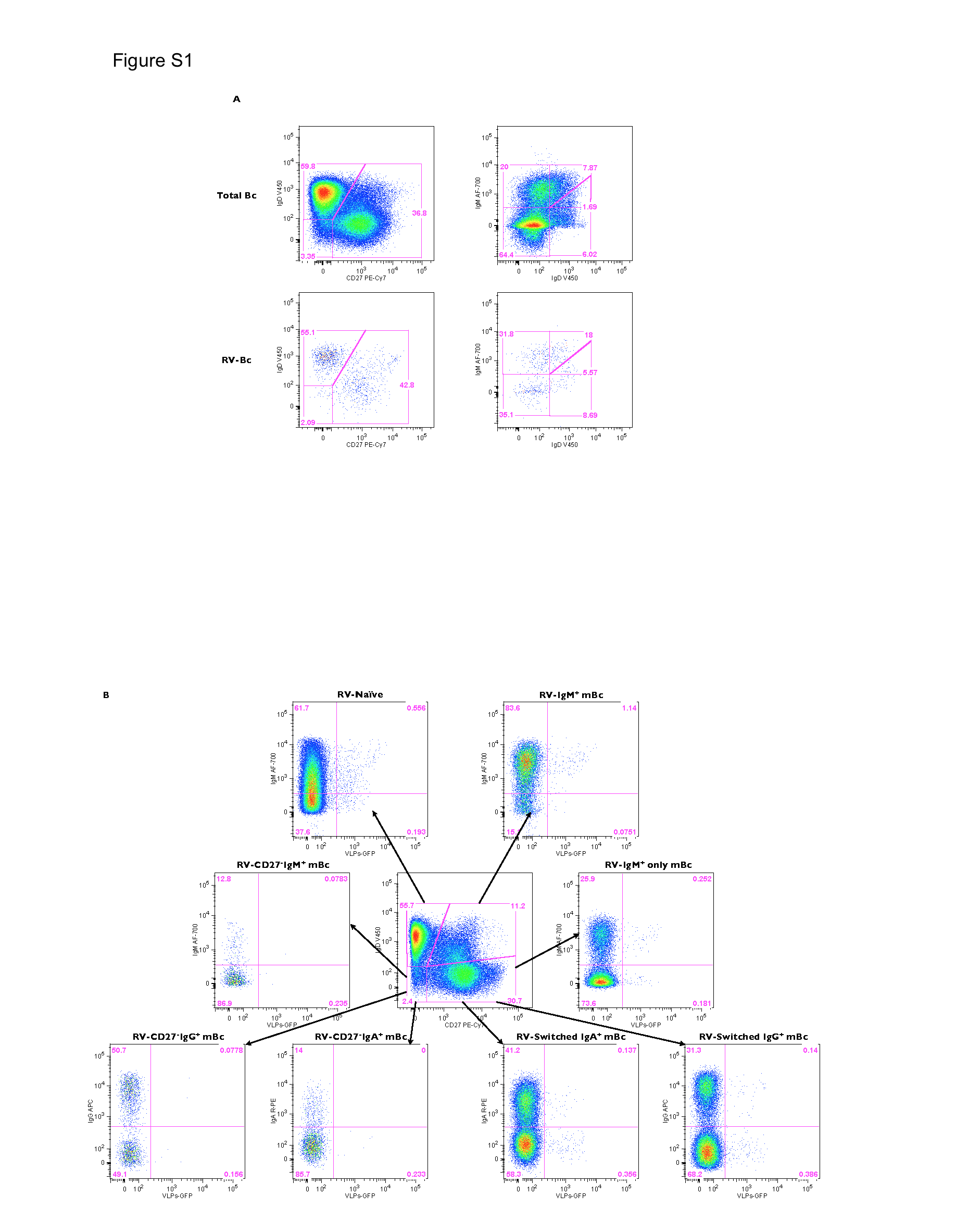

Supplement: Figure S1 — RV-memory B cells gating strategies. Two comparable analysis strategies were used to dissect total and RV-Bc subsets (CD19+) based on their expression of surface markers. A representative result is shown for a HV. Naïve and three main subsets of mBc can be identified based on IgD and CD27 expression: naïve Bc (IgD+CD27−), IgM+ mBc (IgD+CD27+), switched mBc (IgD−CD27+), and CD27− mBc (IgD−CD27−). A. If only CD27+ mBc are considered (naïve and CD27− mBc are excluded) five mBc subsets can be defined in terms of IgM and IgD expression: IgM+ only mBc, IgM+ mBc IgMhiIgDlow, IgM+ mBc IgMlowIgDhi, IgD+ only mBc, and switched mBc (IgM−IgD−). Total (top row plots) and RV-mBc (VLPs-GFP+) (lower row plots) were gated. B. When isotype expression is considered, naïve B cells and IgM+ mBc express IgD and IgM; switched mBc express IgG and IgA, but a subset which only expresses IgM can also be identified on the IgD−CD27+ gate: IgM+ only mBc; and CD27- mBc express IgA, IgG or IgM. (TIFF) [file pone.0097087.s001.tiff]

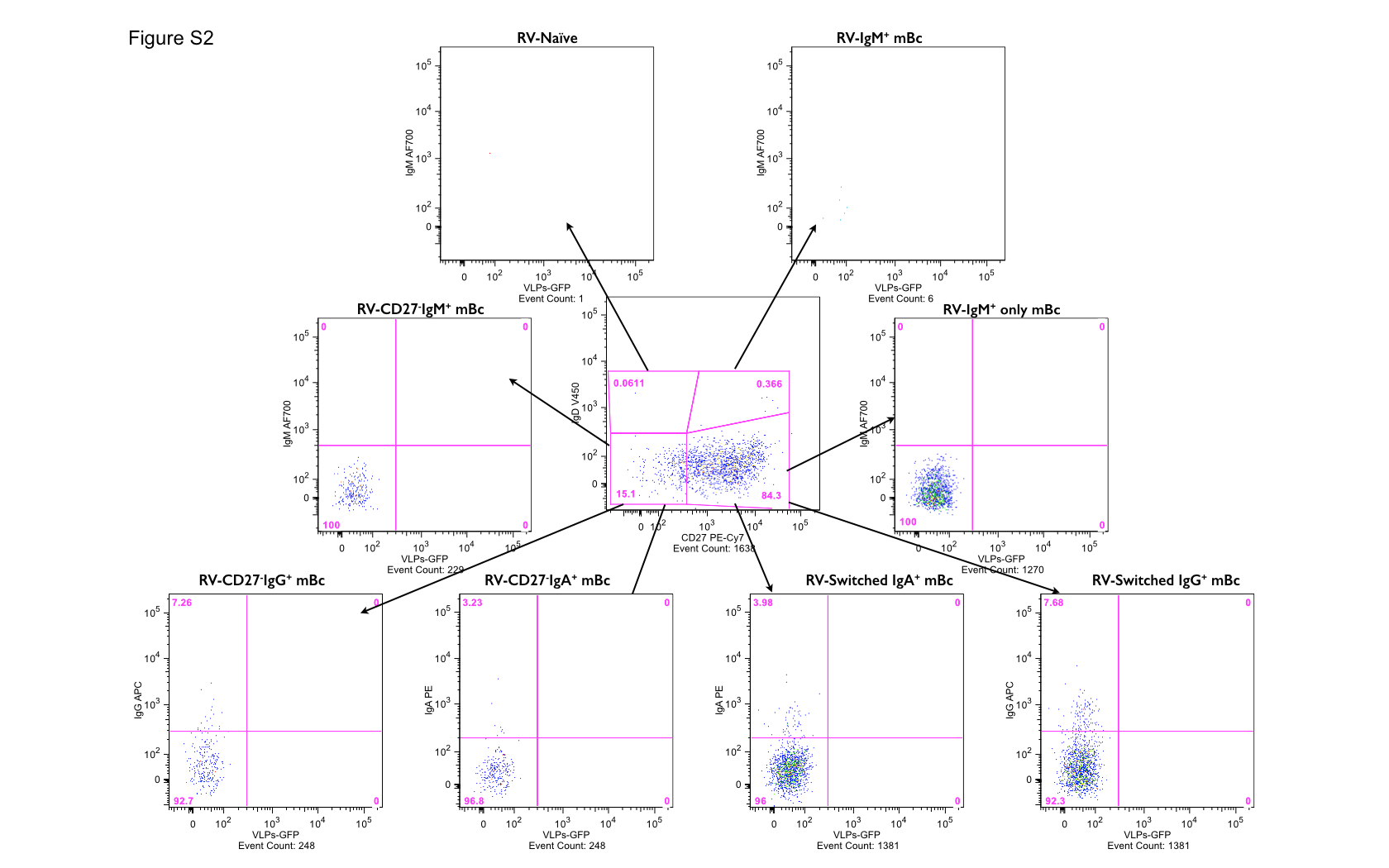

Supplement: Figure S2 — RV-memory B cells gating strategies after RTX treatment. A representative result is shown for a patient four months after RTX treatment. The gating strategy is the same as the one presented in Figure S1B. The number of acquired events per window is shown in addition to the percentage per subset. (TIFF) [file pone.0097087.s002.tiff]

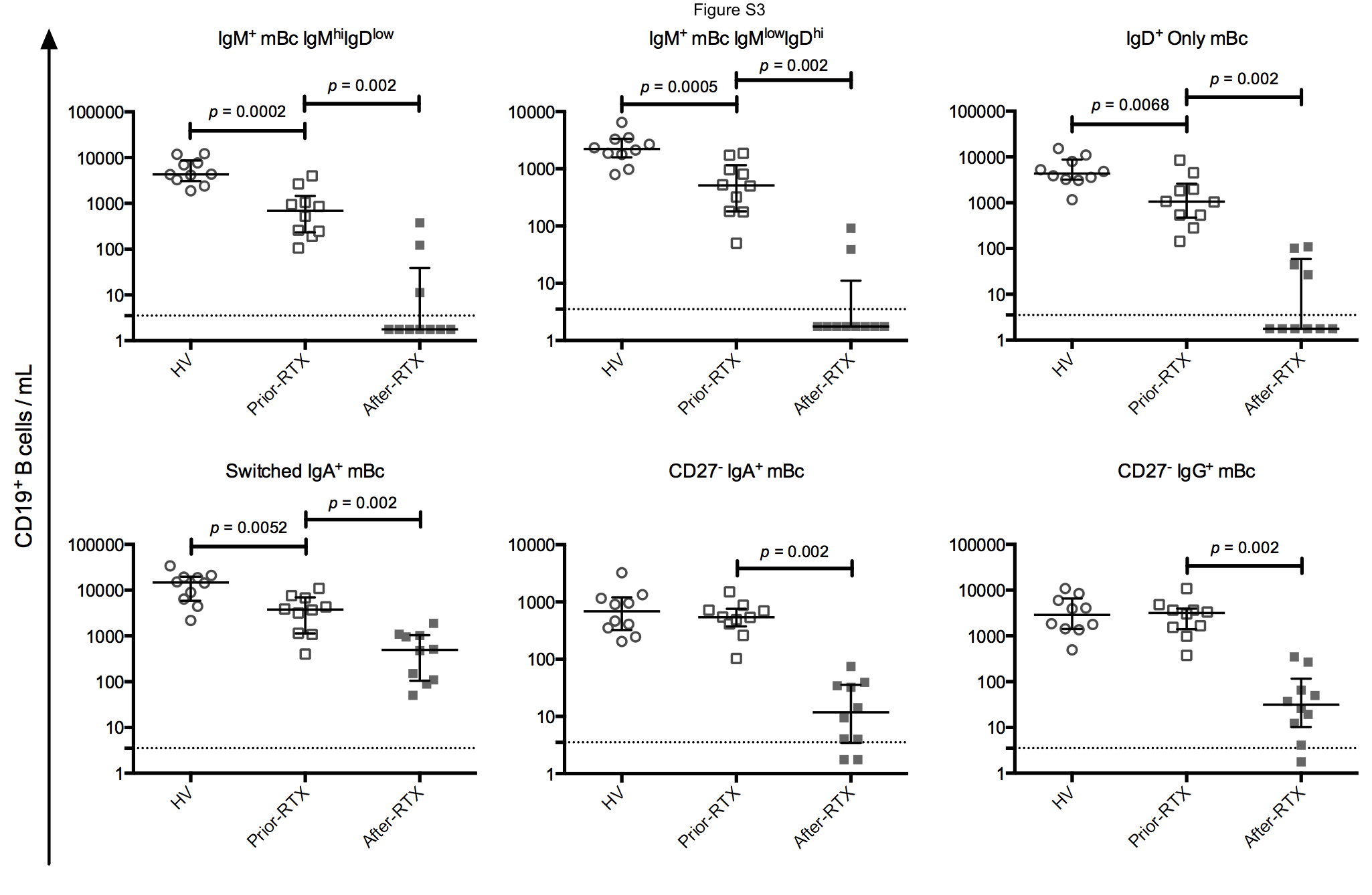

Supplement: Figure S3 — Comparison of other total B cell subpopulations among the study groups. Other total B cell subpopulations studied are shown for healthy volunteers (HV), patients before RTX treatment (prior-RTX) and patients after RTX treatment (after-RTX). The dotted lines represent the estimated flow cytometry detection limit of 3.5 CD19+ B cells/mL. Solid lines and error bars denote the median and interquartile range, respectively. Differences between HV (n = 10) and patients prior-RTX (n = 10) were evaluated with Mann–Whitney tests and between patients prior-RTX and patients after-RTX (n = 10) with Wilcoxon tests. All p values reported are 2-tailed. (TIFF) [file pone.0097087.s003.tiff]

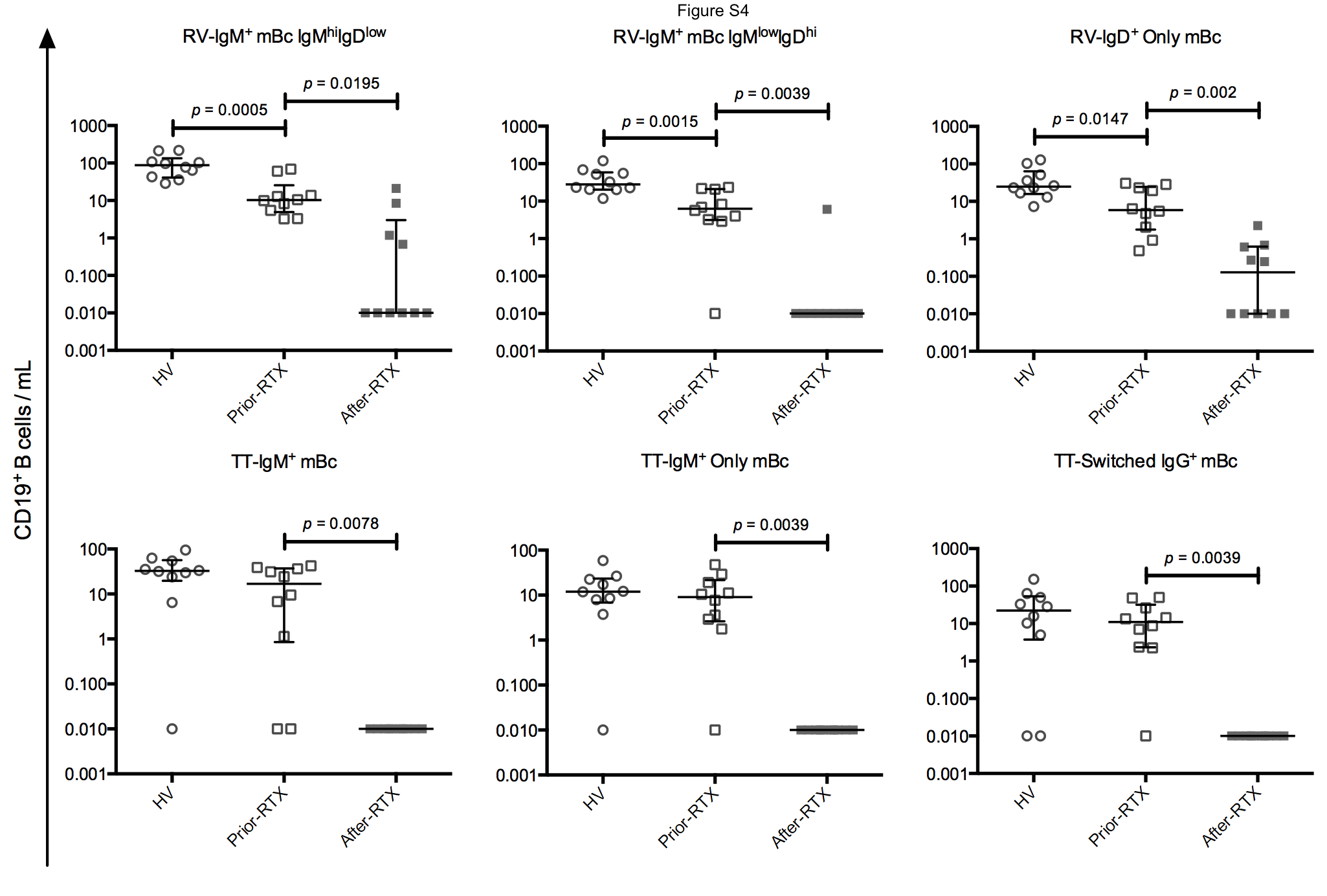

Supplement: Figure S4 — Comparison of other RV and TT-specific B cell subpopulations among the study groups. Other RV- and TT-specific B cell subpopulations studied are shown for HV (n = 10), patients prior-RTX (n = 10) and patients after-RTX (n = 10). Solid lines and error bars denote the median and interquartile range, respectively. Differences between HV and patients prior-RTX were evaluated with Mann–Whitney tests and between patients prior-RTX and patients after-RTX with Wilcoxon tests. All p values reported are 2-tailed. (TIFF) [file pone.0097087.s004.tiff]
