# Supplementary material for: Simultaneous Assessment of Rotavirus-Specific Memory B Cells and Serological Memory after B Cell Depletion Therapy with Rituximab
Source: PLoS One. 2014 May 12;9(5):e97087. doi: 10.1371/journal.pone.0097087 (PMC4018270; doi:10.1371/journal.pone.0097087)
Supplement: Table S1 — Clinical features of patients with autoimmune diseases. (DOCX) [file pone.0097087.s005.docx]

**Table S1. Clinical features of patients with autoimmune diseases**

| **Patient** | **Age** | **Gender** | **Principal diagnosis** | **Concomitant autoimmune disease** | **Disease duration (years)** | **Concomitant immunosuppressive pharmacologic treatment** | **Relevant previous pharmacologic treatment** | **Disease activity before RTX treatment** | **Clinical follow up approximately 6 months after RTX treatment** |
| --- | --- | --- | --- | --- | --- | --- | --- | --- | --- |
| **Pt. 1** | 33 | F | RA | SLE (Lupus nephritis IV AI 9/24) | 6 | PDN, HCQ, AZA, MMF | SSZ, CQ, HCQ, MTX | DAS28: moderate | DAS28: low |
| **Pt. 2** | 69 | F | SLE | Autoimmune thromobocytopenia, Sjögrens’ syndrome, antiphospholipid syndrome, hypothyroidism | 14 | AZA, DFZ | AZA | SLE associated steroid-refractory thrombocytopenia | 60% clinical improvement, platelet count improvement |
| **Pt. 3** | 26 | F | SLE |  | 2 | PDN, HCQ, MMF | MTX | SLEDAI 8: moderate activity, renal compromise | Without clinical improvement. Cyclophosphamide treatment. |
| **Pt. 4** | 33 | F | RA |  | 1 | SSZ, CQ, MTX |  | DAS28: high | DAS28: moderate |
| **Pt. 5** | 35 | F | SLE | Antiphospholipid syndrome, hypothyroidism | 1 | PDN, AZA |  | SLEDAI 6: moderate activity, great articular compromise | Clinical improvement with regard to articular compromise |
| **Pt. 6** | 54 | F | RA | Hypothyroidism | 8 | PDN, MTX | Infliximab | DAS28: high | DAS28: moderate |
| **Pt. 7** | 46 | F | SLE | RA | 3 | MTX, PDN, AZA |  | ND | ND |
| **Pt. 8** | 42 | F | RA | SLE, CREST, hypothyroidism | 7 | PDN, MTX, D-Pen | CQ | DAS28: high | DAS28: moderate |
| **Pt. 9** | 29 | F | SLE | RA | 6 | PDN, AZA, CQ |  |  | SLE without changes |
| **Pt. 10** | 47 | M | RA |  | 2 | MTX, SSZ, CQ |  | DAS28: high | Without clinical improvement |
| **Pt. 11^a^** | 49 | F | RA |  | 3 | PDN, HCQ, MTX, SSZ |  | DAS28: moderate | 60% clinical improvement |
| **Pt. 12** | 58 | F | RA |  | 30 | PDN, HCQ, LEF | Etanercept, MTX | DAS28: moderate | ND |
| **Pt. 13** | 50 | F | RA |  | 3 | DFZ, HCQ, SSZ | Etanercept, LEF | DAS28: moderate | Without clinical improvement |
| **Pt. 14** | 58 | M | RA |  | 3 | PDN, HCQ, MTX |  | DAS28: high | ND |

^a^A complete flow cytometry assay could not be done for patients RTX 1, RTX 13, RTX 20, and RTX 22 at both time frames (before and after RTX treatment); therefore they were not included in the total and Ag-specific Bc analyses.

MTX: Methotrexate, SSZ: Sulfasalazine, HCQ: Hydroxychloroquine, CQ: Chloroquine, PDN: Prednisolone, AZA: Azathioprine, LEF: Leflunomide, MMF: Mycophenolate, Deflazacort: DFZ, D-Penicillamine: D-Pen, ND: not done.
